# Academy of Medicine Notes

**Published:** 1893-08

**Authors:** 


### Academy of Medicine Notes.

DR. C. A. RING has resigned as Secretary of the Section on Surgery, and Dr. F. S. Metcalfe has been elected to fill the position.

---

THE President's address is published in full in this number of the JOURNAL, and deserves to be read and followed by all members of the profession.

---

THE reports of the Secretaries of the various sections show a healthy and gratifying condition of affairs. The outlook for a successful year is very encouraging.

THE election of officers for the Academy resulted as follows: President, Thos. Lothrop, M. D.; Treasurer, Eugene A. Smith, M. D.; Trustee, Frederick W. Bartlett, M. D.

THE Section on Anatomy, Physiology, and Pathology elected the following officers at its last meeting: President, James W. Putnam, M. D.; Secretary, Sidney A. Dunham, M. D.

THE walls of the Academy are adorned with photographs and engravings of some of the former leaders of the local profession. No better place could be found for preserving the records and reminiscences of the profession.

THE Council of the Academy now consists of the following officers: Thomas Lothrop, President; J. A. Pryor, H. E. Hayd, M. A. Crockett, J. W. Putnam, Vice-Presidents; Wm. C. Krauss, Secretary; Eugene A. Smith, Treasurer; Frederick W. Bartlett, Roswell Park, Alphonse Dagenais, Trustees; H. Dowd, Secretary Section on Medicine; F. S. Metcalfe, Secretary Section on Surgery; S. A. Dunham, Secretary Section on Pathology; L. C. Randall, Secretary Section on Obstetrics.

